# Characterization of microbial antifreeze protein with intermediate activity suggests that a bound-water network is essential for hyperactivity

**DOI:** 10.1038/s41598-021-85559-x

**Published:** 2021-03-16

**Authors:** N. M.-Mofiz Uddin Khan, Tatsuya Arai, Sakae Tsuda, Hidemasa Kondo

**Affiliations:** 1 Graduate School of Life Science, Hokkaido University, Sapporo, 060-0810 Japan; 2 Department of Chemistry, Dhaka University of Engineering and Technology, Gazipur Gazipur, 1700 Bangladesh; 3 Bioproduction Research Institute, National Institute of Advanced Industrial Science and Technology (AIST), 2-17-2-1, Tsukisamu-Higashi, Toyohira, Sapporo, Hokkaido 062-8517 Japan; 4 OPERANDO Open Innovation Laboratory, National Institute of Advanced Industrial Science and Technology (AIST), Tsukuba, 305-8563 Japan

**Keywords:** Structural biology, Biomaterials - proteins

## Abstract

Antifreeze proteins (AFPs) inhibit ice growth by adsorbing onto specific ice planes. Microbial AFPs show diverse antifreeze activity and ice plane specificity, while sharing a common molecular scaffold. To probe the molecular mechanisms responsible for AFP activity, we here characterized the antifreeze activity and crystal structure of *Tis*AFP7 from the snow mold fungus *Typhula ishikariensis*. *Tis*AFP7 exhibited intermediate activity, with the ability to bind the basal plane, compared with a hyperactive isoform *Tis*AFP8 and a moderately active isoform *Tis*AFP6. Analysis of the *Tis*AFP7 crystal structure revealed a bound-water network arranged in a zigzag pattern on the surface of the protein’s ice-binding site (IBS). While the three AFP isoforms shared the water network pattern, the network on *Tis*AFP7 IBS was not extensive, which was likely related to its intermediate activity. Analysis of the *Tis*AFP7 crystal structure also revealed the presence of additional water molecules that form a ring-like network surrounding the hydrophobic side chain of a crucial IBS phenylalanine, which might be responsible for the increased adsorption of AFP molecule onto the basal plane. Based on these observations, we propose that the extended water network and hydrophobic hydration at IBS together determine the *Tis*AFP activity.

## Introduction

Organisms inhabiting cold regions have developed diverse strategies to prevent freeze damage. Antifreeze proteins (AFP) are part of one such strategy. These proteins are structurally diverse and produced by various organisms, including fish^1^, insects^2^, plants^3^, and microbes^4^.

AFPs have specific affinity for a single ice crystal, to arrest its growth in the direction normal to the bound surface. They noncolligatively depress the non-equilibrium freezing point (*T*_f_) of a solution below the melting point (*T*_m_)^5,6^ via Gibbs–Thomson effect^7^. The difference between *T*_m_ and non-equilibrium *T*_f_ is called thermal hysteresis (TH), which is a prime indicator of the AFP antifreeze activity^6–9^. Based on the TH value, AFPs are classified into two main groups, namely, moderately active and hyperactive AFPs. Hyperactive AFPs achieve up to 2–5 °C TH, which is 10–100 times that of the moderately active AFPs at identical protein concentrations^10^. Hyperactivity can be ascribed to the binding affinity to the basal plane of ice as well as prism and pyramidal planes, leading to rapid ice growth (burst) below *T*_f_, and expansion perpendicular to the ice *c*-axis with a dendritic pattern with a sixfold symmetry^9,11^. By contrast, typical moderately active AFPs bind to prism and/or pyramidal planes to form a bipyramidal ice between *T*_m_ and the non-equilibrium *T*_f_ (defined as the TH gap). Below the non-equilibrium *T*_f_, the ice crystal bursts at the two unbound bipyramid tips, which lie along the *c*-axis, forming a needle-shaped ice^9,12–14^. The specific affinity toward sets of water molecules constituting discrete ice surfaces is attributable to the ice-binding site (IBS), localized on the flat surface of an AFP molecule^8,9^. IBS in various AFPs show vast amino acid sequence and structure diversity, which implies that each AFP evolved from a different ancestor molecule to adapt to the cold environment by acquiring ice-binding ability^9^. Therefore, understanding the detailed molecular mechanism that defines ice-binding specificity is crucial for the elucidation of adaptation to cold environment associated with AFP evolution.

Most microbial AFPs, produced by such microorganisms as fungi^4,15^, diatoms^16^, and bacteria^17^, belong to a widespread family formerly designated as “domain of unknown function” (DUF) 3494^18^ in the Pfam database^19^, and currently categorized as “ice-binding–like family”. Characterization of DUF3494 AFPs (or ice-binding proteins; IBPs) revealed their highly diversified antifreeze activities. For example, AFPs from *Colwellia sp.* (*Col*AFP)^20^, *Flavobacterium frigoris* PS1 (FfIBP)^21^, *Typhula ishikariensis* isoform 8 (*Tis*AFP8)^22^, and *Shewanella frigidimarina* (*Sf*IBP_1)^23^ are hyperactive, whereas those from *Leucosporidium* sp. (*Le*IBP)^24^, *T. ishikariensis* isoform 6 (*Tis*AFP6)^25^, *Antarctomyces psychrotrophicus* (*Anp*IBP1a)^26^, and *Fragilariopsis cylindrus* (*Fc*IBP)^16^ show moderate activity. X-ray crystallographic studies revealed that all these AFPs adopt a similar β-helical structure. The putative IBS of this protein family is located on the flat surface of one β-helix^20,22,25^. The level of IBS sequence identity shared by various AFPs is similar to that shared by other regions of these proteins; however, no conserved sequence motifs in IBS have been identified. Based on phylogenetic analysis, microbial AFP genes are presumably propagated via horizontal gene transfer^26,27^. Less is known, however, about the key determinants of hyperactivity and moderate activity of this AFP family.

Three-dimensional structural analyses of hyperactive AFPs from arthropods and bacteria have revealed unique features of the IBS and bound water structure. For instance, insect AFPs form a β-helical structure with repetitive sequence motifs. Conserved residues in these motifs (Thr–X–Thr, where X is any amino acid) are organized into two parallel arrays along the β-helical axis, constructing the IBS on one flat face of the β-helix^28,29^. On the array, Thr residues are aligned at constant intervals, matching the distance between the water molecules on the ice plane^28^. Further, the hydroxy groups in the side chain of IBS Thr residues anchor the bound water molecules at constant intervals, which match the basal and prism plane of the ice surface^29^. According to a molecular dynamics simulation study, regularly ordered water clathrate gives rise to the highest affinity for the ice basal plane^30^. Crystal structure analysis of *Mp*AFP_RIV from the Antarctic bacterium *Marinomonas primoryensis* revealed an array of ordered water molecules aligned along the IBS residues. The bound water molecules are anchored to a repetitive motif of IBS residues (Thr–Gly–Asn/Asp) to form ice-like structure, which led to the proposal of the “anchored clathrate water” mechanism for AFP–ice interaction^31^.

IBS of microbial AFPs with the DUF3494 fold lack the repetitive amino acid sequence and consensus motif^20,23,25^, which implies that a different architecture drives their hyperactivity. Based on studies of *Col*AFP and *Tis*AFP8, Hanada et al.^20^ and Cheng et al*.*^22^, respectively, proposed that the b-face of IBS be subdivided into the β-sheet and adjacent loop regions, termed a compound IBS, which stick to the discrete ice plane. Based on the crystal structure analysis and ice-docking modeling combined with a mutation study, IBS loop residues were proposed as crucial for the affinity to the basal plane, conferring hyperactivity^20,22^. Nevertheless, it is still not clear how the compound IBS recognizes the specific ice plane.

The snow mold fungus *T. ishikariensis* secretes AFP (*Tis*AFP) at 0 °C^15^. Culture filtrates contain seven AFP isoforms (*Tis*AFP2–8), 223 amino acid residues each. We have previously shown that *Tis*AFP8 is hyperactive^22^ whereas *Tis*AFP6 is only moderately active^25^, despite high sequence identity (83%) shared with *Tis*AFP8. Another isoform, *Tis*AFP7, shares an even higher identity with *Tis*AFP8 (91%) and *Tis*AFP6 (87%). We reasoned that *Tis*AFP7 might be a suitable target molecule for gaining insight into the relationship between AFP antifreeze activity and IBS structure. In the current study, we characterized *Tis*AFP7 using TH measurements, ice crystal morphology observation, and visualization of AFP-bound ice planes by fluorescence-based ice plane affinity (FIPA) analysis. We also analyzed the crystal structures of *Tis*AFP7 and its defective mutants. The analysis revealed a distinct bound-water network on IBS, which presumably defines the binding affinity of microbial AFPs for the basal plane. The findings broaden the understanding of cold-adaptation mechanism of psychrophilic and cold-tolerant microorganisms, and will inform the design of artificial compounds with antifreeze properties.

## Results and discussion

### *Tis*AFP7 shows intermediate TH activity that falls between that of *Tis*AFP6 and *Tis*AFP8

**Figure 1:**
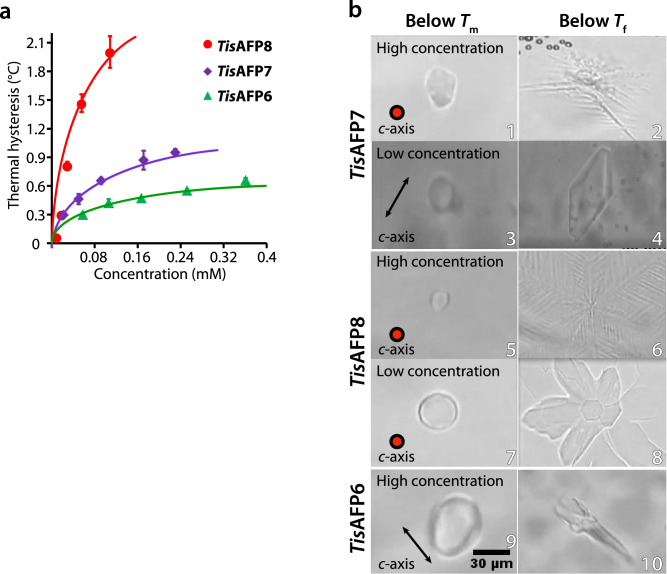
Comparison of the antifreeze properties of *Tis*AFP7 and two other *Tis*AFP isoforms, the moderately active* Tis*AFP6 and hyperactive* Tis*AFP8. (**a**) Plot of thermal hysteresis values for the three isoforms at various protein concentrations. Purple, *Tis*AFP7; red, *Tis*AFP8; and green, *Tis*AFP6. The values are means and the error bars are standard deviations from three independent observations at each protein concentration. The values for *Tis*AFP8 and *Tis*AFP6 are adopted from reference^22^. The curves were fitted for ease of interpretation. (**b**) Microscopic images of ice crystal morphology in solution of AFP isoforms at melting (*T*_m_) and non-equilibrium freezing temperature (*T*_f_). High and low concentrations denote 0.23 mM and 0.01 mM for *Tis*AFP7; and 0.12 mM and 0.01 mM for* Tis*AFP8. For *Tis*AFP6, ice crystals grown at 0.1 mM are shown. The *c*-axis direction of the ice crystal is shown in the figure as a circle or an arrow. The images are representative of three observations under each condition.

Antifreeze activity of *Tis*AFP7 was first evaluated by TH measurements, at a series of protein concentrations (0.022–0.23 mM; Fig. 1a). TH value increased with an increasing protein concentration with a maximum of 0.95 °C at 0.23 mM protein. We have previously characterized TH activity of the hyperactive isoform *Tis*AFP8^22^ and the moderately active isoform *Tis*AFP6^25^. *Tis*AFP8 showed the highest TH (approximately 2 °C) at 0.11 mM, and *Tis*AFP6 exhibited the lowest TH (approximately 0.6 °C) at 0.35 mM. Comparing the TH values of these AFPs at the same protein concentration, e.g., 0.11 mM, *Tis*AFP7 TH was approximately 35% that of *Tis*AFP8, and 1.15-fold that of *Tis*AFP6. Based on the TH values, the antifreeze activity of the three isoforms could be ordered as *Tis*AFP8 > *Tis*AFP7 > *Tis*AFP6 (highest to lowest). *Tis*AFP7 shares 91% and 87% sequence identity with *Tis*AFP8 and *Tis*AFP6, respectively, which supports its intermediate position among the isoforms. Based on the TH value, *Tis*AFP7 is an intermediately active AFP. The denaturation profile monitored by CD spectra from 20 to 70 °C is shown in Supplementary Fig. S3c. Denaturation temperature (*T*_m_) was estimated as a midpoint of the profile as 47.5, 50.0, 53.5 °C for *Tis*AFP7, *Tis*AFP8, and *Tis*AFP6, respectively. Besides, defective mutants of *Tis*AFP7 exhibited no change in their *T*m, as shown in Supplementary Fig. S3d. Our current result for *T*_m_ shows that the order of the thermal stability is not correlated with that of TH values. Contrary to our results, higher *T*_m_ was reported for moderately active *Le*IBP^21^ (61.0 °C) and *Efc*IBP^32^ (66.4 ± 2.7 °C), and lower *T*_m_ was reported for hyperactive *Ff*IBP^21^ (56.4 °C) and IBPv^33^ (53.5 °C). On the other hand, thermal denaturation experiments for chimeric proteins for *Le*IBP and *Ff*IBP^21^ demonstrated that denaturation temperature of microbial AFP might be affected by various structural segments including capping head regions, which cover both ends of β-helix. Therefore, the authors of the referenced paper 21 suggested that *T*_m_ is not directly related to antifreeze activity. For *Tis*AFP6, most of the replaced residues (7 residues out of 11) from *Tis*AFP7 and *Tis*AFP8 are situated in the capping head regions, which seems to originate the highest *T*m among the isoforms.

### *Tis*AFP7 exhibits a concentration-dependent ice-bursting pattern

Figure 1b presents microscopic images of a cooled ice crystal used for TH measurements. At 0.23 mM *Tis*AFP7 (TH 0.9 °C), the single ice crystal retained its original size below *T*_m_ (Fig. 1b, panel 1). Once the temperature reached the non-equilibrium *T*_f_, the ice crystal rapidly grew (burst) perpendicular to the *c*-axis, with a hexagonal-stellar pattern with dendritic branches (Fig. 1b, panel 2), which is typically observed for hyperactive AFPs^22,23^. The observed restriction of ice growth along the *c*-axis could be ascribed to AFP binding to the basal plane of ice crystal, as proposed by Scotter et al.^10^, a hallmark of hyperactive AFP. We showed that *Tis*AFP7 bound to the basal plane at high concentration (0.23 mM). At a relatively low concentration (0.01 mM), the seed ice crystal changed shape to a hexagonal bipyramid with truncated tips in the TH gap, and then grew rapidly, maintaining the bipyramidal shape (Fig. 1b, panels 3 and 4). This indicated that *Tis*AFP7 affects the ice morphology and ice-bursting pattern in a concentration-dependent manner. The ice morphology at a low protein concentration revealed that the *c*-axis growth was not as inhibited as at a high protein concentration. The known hyperactive AFPs inhibit *c*-axis ice growth, with a hexagonal dendritic bursting pattern even at a low protein concentration. For example, the hyperactive isoform *Tis*AFP8 supports a hexagonal pattern burst at high and low concentrations (0.12 mM and 0.01 mM), as shown in Fig. 1b, panels 5, 6, 7, and 8. A similar bursting pattern was reported for sbwAFP (*Choristoneura fumiferana*)^34^ and SfIBP_1^23^. On the other hand, *Tis*AFP6 supported ice bursting along the *c*-axis even at a high protein concentration (0.1 mM; Fig. 1b, panels 9 and 10), which has also been reported for typical moderately active AFPs, including fish type III^35^ and type II^36^ proteins. Comparison of the ice-bursting pattern of *Tis*AFP7 with those of the known AFPs suggests that it exhibits intermediate activity, positioned between those of hyperactive and moderately active AFP species.

### FIPA analysis reveals that *Tis*AFP7 adsorbs to entire planes of ice crystal

**Figure 2:**
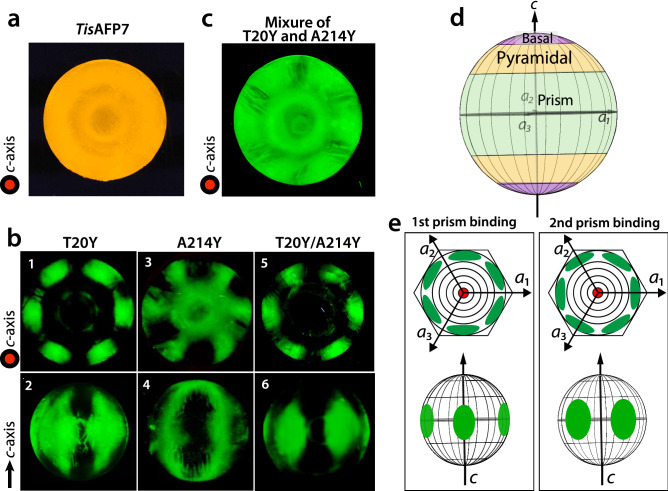
Ice plane specificity of wild-type *Tis*AFP7 and its mutants, as determined by FIPA analysis. The concentration of protein solution was 0.007 mg/ml in all experiments. (**a**) Fluorescence image of a single ice-crystal hemisphere grown in a solution of wild-type *Tis*AFP7 labeled with an orange fluorescent dye. The single ice crystal was mounted on the cold finger perpendicular to the basal plane. The *c*-axis direction of the ice crystal is shown in the figure as a circle. (**b**) Fluorescence from single ice-crystal hemispheres grown in solutions of *Tis*AFP7 T20Y, A214Y, and T20Y/A214Y mutants labeled with a green fluorescent dye. Upper panels, the hemispheres were mounted in the same orientation as in (**a**); lower panels, the hemispheres were mounted with a primary prism plane perpendicular to the cold finger. The *c*-axis direction is shown as an arrow. (**c**) A single ice crystal grown in the solution containing equal concentrations of T20Y and A214Y mutants (0.0035 mg/ml of each). (**d**) Schematic illustration of ice planes mapped onto a hemispherical-shaped ice crystal. The polar regions correspond to the ice basal plane. The equator and middle latitude zones correspond to the prism and pyramidal planes, respectively. (**e**) Schematic illustrations of fluorescent patches corresponding to the primary and secondary prism planes on the ice hemisphere in known orientation. When the ice crystal is mounted with the basal plane perpendicular to the cold finger, the primary (1st) prism plane appears between the *a*_1_-, *a*_2_-, and *a*_3_-axes with a hexagonal symmetry, as shown by the upper images. The secondary (2nd) prism plane is situated on the region pierced with the *a*_1_-, *a*_2_-, and *a*_3_-axes. When the ice crystal is mounted with the primary prism plane perpendicular to the cold finger, three patches are observed on the equator of the hemisphere for the primary prism plane, whereas two patches are seen for the secondary prism plane.

To visualize the AFP-bound planes of ice crystal (Fig. 2), we prepared fluorescently-labeled *Tis*AFP7 and evaluated it by FIPA analysis. Figure 2a shows a fluorescent image of a grown ice hemisphere mounted on the cold finger parallel to the *c*-axis of the ice crystal. The orange fluorescence of the entire hemisphere indicated that *Tis*AFP7 was incorporated in the entire ice structure, i.e., that all ice planes, including the basal plane, were covered with *Tis*AFP7. Similar observations have been reported for hyperactive AFPs, including *Tis*AFP8^22^, *Col*AFP^20^ *Mp*AFP_RIV^31^, sbwAFP (*C. fumiferana*)^37^, and *Tm*AFP (*Tenebrio molitor*)^37^. Some moderately active AFPs, when assayed at a relatively high concentration (0.1 mg/ml), also exhibit whole-hemisphere binding, e.g., BpAFP^14^ and AFPII^38^ (*Brachyopsis rostratus*), with a concentration-dependent multiple-plane binding. In the current study, FIPA analysis of *Tis*AFP7 was performed using a low protein concentration (0.007 mg/ml); the protein’s multiple-plane binding ability was higher than those of the typical moderately active AFPs. The ability to bind the basal plane has been also reported for moderately active AFPs, such as *Tis*AFP6^25^ and *Lp*IBP^39^ (*Lolium perenne*), but with the basal and primary prism planes covered by separate hemisphere protein patches, which was different from the whole-plane binding observed for *Tis*AFP7. Based on the above, we propose that *Tis*AFP7 binds all ice planes, including the basal plane, with an intermediate binding strength, positioned between those of hyperactive and moderately active AFP. This is a unique ice-binding property, reflecting the sequence identity shared by the hyperactive and moderately active *Tis*AFP isoforms.

### Crystal structure of *Tis*AFP7

Diffraction data for wild-type *Tis*AFP7 was collected using a crystal grown as a thin plate-like shape. The crystal belongs to the orthorhombic space group *P*2_1_2_1_2_1_, with unit cell parameters of *a* = 57.12 Å, *b* = 62.99 Å, and *c* = 101.21 Å, with two molecules in an asymmetrical unit. The crystal structure of *Tis*AFP7 was determined at 1.54 Å resolution by a molecular replacement method. The statistics for data collection and refinement are summarized in Table 1. The final structure was refined with *R* factor of 0.195 and Free *R* factor of 0.229, and was composed of 446 (223 × 2) residues and 746 solvent molecules. The refined *Tis*AFP7 structure exhibited the root-mean–squared deviation (RMSD) of 0.37 Å and 0.34 Å from Cα atoms of *Tis*AFP6 and *Tis*AFP8, respectively, reflecting high sequence identities shared by the isoforms.

**Table 1:** Data collection and refinement statistics for *Tis*AFP7 and *Tis*AFP7 T20Y.

Crystal	*Tis*AFP7	*Tis*AFP7 T20Y
**Data collection**		
Space group	*P*2_1_2_1_2_1_	
Unit cell parameters (*a*, *b*, *c*), (**Å**)	57.12, 62.99, 101.21	56.79, 63.14, 102.09
Beam line	Photon factory BL-1A	
Wavelength (Å)	1.1000	
Resolution range (Å)	49.75–1.54	49.68–1.72
*R*_merge_^a^^,^^b^	0.131 (1.167)	0.192 (1.137)
Observed reflections	689,607	530,653
Independent reflections	54,671	41,109
Completeness^a^ (%)	99.8 (99.0)	99.8 (99.9)
Multiplicity^a^	12.6 (13.1)	12.9 (12.6)
< *I*/σ(*I*) > ^a^	12.3 (2.3)	8.6 (2.3)
**Refinement**		
*R* factor^a^^,^^c^	0.195 (0.457)	0.208 (0.398)
Free *R* factor^a^^,^^c^^,^^d^	0.229 (0.467)	0.246 (0.440)
R.M.S. bond length (Å)	0.013	0.010
R.M.S. bond angle (°)	1.709	1.633
Residues	446 (223 × 2)	446 (223 × 2)
**Number of non-hydrogen protein atoms**		
Protein	3131	3120
Water	746	554
Other	30 (SO_4_^2−^ × 6)	3 (Mg^2+^ × 3)
**Ramachandran plot**^**e**^** (%)**		
Residues in favored regions	96.4	97.5
Residues in allowed regions	3.6	2.5
Residues in outliner regions	0	0
Average B factor (Å^2^)	11.0	12.0

^a^Values in parentheses are for the highest-resolution shell.

^b^*R*_merge_ = $\sum {\sum }_{j}\left|\langle I\left(h\right)\rangle -I{\left(h\right)}_{j}\right|\sum {\sum }_{j}\langle I\left(h\right)\rangle$, where $\langle I\left(h\right)\rangle$ is the mean intensity of a set of equivalent reflections.

^c^*R* factor = $\sum \left|\left|{F}_{\mathrm{obs}}\left(h\right)\right|-\left|{F}_{\mathrm{calc}}\left(h\right)\right|\right|\sum \left|{F}_{\mathrm{obs}}\left(h\right)\right|$, where ${F}_{\mathrm{obs}}$ and ${F}_{\mathrm{calc}}$ are the observed and calculated structure factors, respectively.

^d^For the calculation, 5% of the data were randomly chosen and the free *R* factor calculated^58^.

^e^Statistics were obtained from MolProbity^59^.

**Figure 3:**
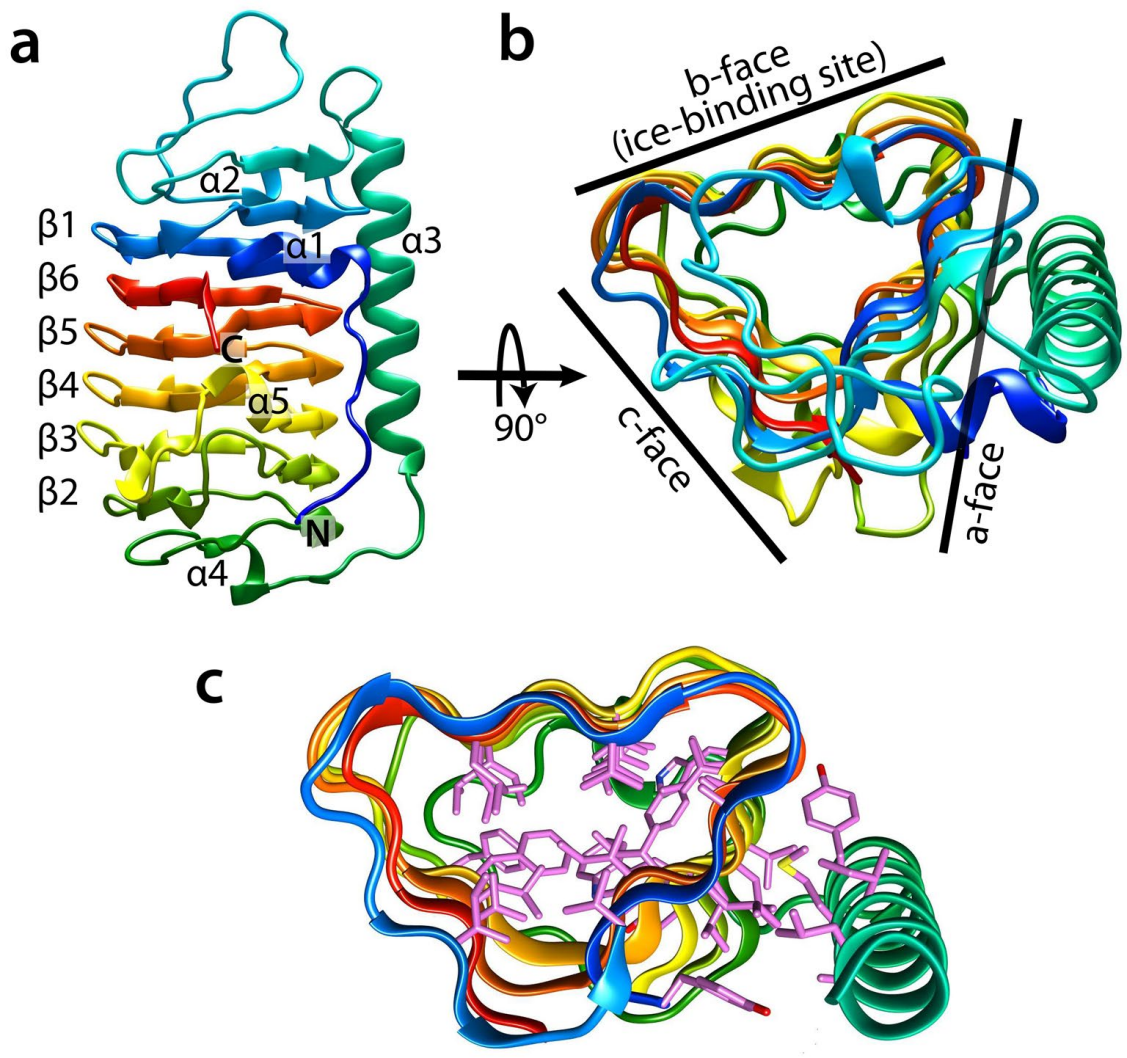
Schematic illustration of the *Tis*AFP7 crystal structure. (**a**) View along the normal to the β-helical axis with the N- and C-termini denoted in blue and red, respectively (the helical axis runs in parallel to the page, from top to bottom, and the molecule is viewed along the axis perpendicular to the helical axis). Helical coils of irregular length are denoted as β-1, and β-6 to β-2, from N-terminal half of the solenoid. The long α-helix is denoted as α-3, with additional 3_10_ helices numbered α-1, α-2, α-4, and α-5. (**b**) View along the β-helical axis upon 90° rotation of the view in (**a**), showing a triangular cross-section (the helical axis is perpendicular to the page). The molecular faces of β-helix are denoted as the a-, b-, and c-faces, accordingly. Here, the c-face is a putative ice-binding site. (**c**) Hydrophobic core formed within the β-helices, and between the a-face and α-3 helix, drawn as a stick model. The *Tis*AFP7 structures presented in this and other figures were prepared using UCSF Chimera^66^.

Figure 3 shows a schematic representation of *Tis*AFP7. The structure is dominated by a right-handed β-helical domain, a typical DUF3494 structure (Fig. 3a). The β-helical domain is composed of 184 residues with six helical coils (β1–β6), which constitute the N-terminal portion of the β-helix (Ile16–Gly73) and C-terminal portion of the β-helix (Ser98–Lys223). The helical coils are ordered in the sequence β1-β6-β5-β4-β3-β2, which is unusual for typical β-helical proteins, and unique for DUF3494. Further, β1 is composed of 17 residues (Ile16–Gly32); β6 is composed of 18 residues (Arg206–Lys223); β5 is composed of 18 residues (Val188–Gly205); β4 is composed of 21 residues (Lys167–Gly187); β3 is composed of 28 residues (Thr139–Ala166); and β2 is composed of 22 residues (Pro117–Ser138). The non-uniform numbers of residues in each coil (17–28 residues) result in a slightly swelled backbone structure at an end of the domain, akin to a semi-pear shape. An additional long α-helix (Thr74–Arg97) is situated parallel to the β-helix. The N-terminal segment (Ala1–Leu8) is extended in an antiparallel orientation with respect to the α-helix. Four short segments of 3_10_ helices (α1, Gly9–Asn13; α2, Gly41–Phe43; α4, Ile105–Gly108; and α5, Ala166–Asn168) connect the β-strands and loop segments. Figure 3b shows *Tis*AFP7 viewed along the β-helix axis. A triangular cross section is seen, with three flat surfaces (a-, b-, and c-face) composed of parallel β-sheets. The a-face is covered with a long α-helix and the N-terminal segment, while the b- and c-faces are exposed to the solvent. In Fig. 3c, the hydrophobic residues facing the inside of the β-helix and the interface between the a-face and long α-helix are shown, and form the hydrophobic cores of the molecule.

### Ice-plane specificity of the loop region and β-sheet of IBS

**Figure 4:**
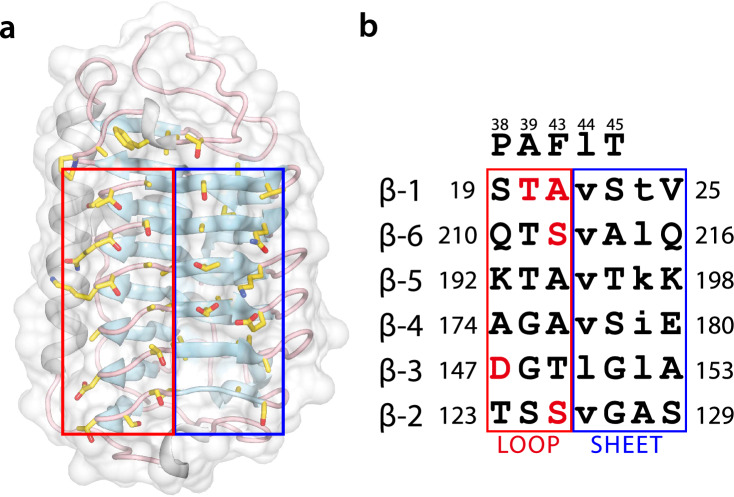
Front view of the putative ice-binding site (IBS) of *Tis*AFP7, viewed from the b-face. The loop and sheet regions of the compound IBS are enclosed by red and blue boxes, respectively. In (**a**), the side chains of residues composing the putative IBS are drawn as a stick model. In (**b**), amino acids at corresponding positions in IBS are shown. Upper and lower letters indicate outward- and inward-facing residues, respectively. Five residues that are substituted from IBS of *Tis*AFP8 are indicated by red letters.

Figure 4 shows the putative IBS of *Tis*AFP7, which is located on the flat b-face of the β-helix. IBS consists of 26 residues of a six-stranded parallel β-sheet and 21 residues of the adjacent loop region connected to the a-face of the β-helix. Only five residues differentiate IBS of *Tis*AFP7 from the hyperactive *Tis*AFP8 isoform, all of which are located in the loop region. To assess their role in ice binding, Thr20 in the loop region, reported to be one of the residues key for *Tis*AFP8 hyperactivity^22^, was replaced with Tyr. The crystal structure of *Tis*AFP7 T20Y was determined at 1.72 Å resolution. The crystal was isomorphous with the wild-type crystal, and the final structure was refined with R-factor of 0.208 and Free R of 0.246, containing 446 residues and 554 solvent molecules.

**Figure 5:**
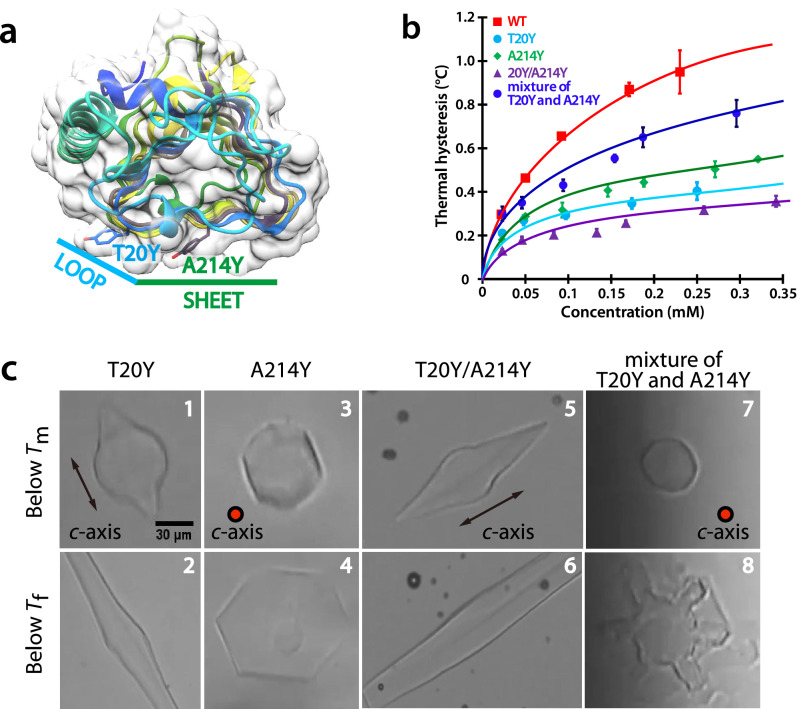
Effect of amino acid substitution on ice-binding site (IBS) in *Tis*AFP7. (**a**) Locations of the T20Y and A214Y point substitutions in the loop and sheet regions of IBS, respectively. (**b**) Thermal hysteresis profiles as a function of protein concentrations. The values are means and the error bars are standard deviations from three independent observations at each protein concentration. (**c**) Microscopic images of single ice crystals in the sample solutions. Upper panels, ice crystal morphology in the thermal hysteresis gap (below the melting point). Lower panels, ice crystal morphology below the non-equilibrium freezing point. The *c*-axis direction of the ice crystal is shown in the figure as a circle or an arrow. The images are representative of three observations under each condition.

Another sampled substitution site was Ala214, located at the center of the IBS β-sheet. The two sites are shown in Fig. 5a. We predicted that the substitutions would interfere with the ice binding because of the steric hindrance of the bulky side chain of Tyr. Indeed, both substitutions resulted in a reduced TH activity (0.4 °C at 0.25 mM T20Y mutant and 0.45 °C at 0.27 mM A214Y mutant), equivalent to a 60% and 50% reduction from wild-type *Tis*AFP7, accordingly (Fig. 5b). In the TH gap of the T20Y mutant, very small ice tips protruded from the seed ice crystal (Fig. 5c, panel 1). At the moment of freezing, the ice crystal rapidly grew in two directions along the *c*-axis (Fig. 5c, panel 2). A similar growth pattern was observed for wild-type *Tis*AFP7 at a low protein concentration (0.01 mM, Fig. 1b, panels 3 and 4) and *Tis*AFP6 (Fig. 1b, panels 9 and 10), which suggested that the T20Y substitution reduced the protein’s ability to bind the basal plane. In the case of the A214Y mutant, the ice crystal was modified to form a rounded hexagonal plate below *T*_m_ (Fig. 5c, panel 3) and expanded maintaining this shape (Fig. 5c, panel 4). Hence, the A214Y mutant retained the affinity for the basal plane. The TH of a double T20Y/A214Y mutant was lower than those of single mutants, and the mutant exhibited an impaired ice-growth inhibition (Fig. 5c, panels 5 and 6). On the other hand, a mixture of equal concentrations of the two single mutants recovered the TH to 68% that of the wild type (Fig. 5b). Furthermore, ice morphology in the mixture was the same as that for wild-type *Tis*AFP7 (Fig. 5c, panels 7 and 8).

Next, the single mutants T20Y and A214Y, and double mutant T20Y/A214Y were fluorescently labeled and subjected to FIPA analysis (Fig. 2b, panels 1, 3, and 5). The samples were mounted on the cold finger in the same orientation as that for wild-type *Tis*AFP7 analysis shown in Fig. 2a. In this orientation, the basal plane was illuminated in the center zone of the hemisphere, and the perimeter zones corresponded to the primary or secondary prism planes with a hexagonal symmetry, as schematically shown in Fig. 2e (upper panels). In Fig. 2b, panels 2, 4, and 6, the hemispheres were mounted with the primary prism plane perpendicular to the cold finger. Figures 2d,e (bottom panels) show a schematic overview of the corresponding regions of the prism, pyramidal, and basal planes in the equator, middle latitude, and polar region of the hemisphere, respectively. As shown in Fig. 2b, panels 1 and 2, the T20Y mutant covered only the perimeter zone of the hemisphere, corresponding to the secondary prism planes. The reduced coverage of ice planes by the T20Y mutant indicated lost ice-binding affinity for the basal, primary prism, and some pyramidal planes. On the other hand, the A214Y mutant retained the ability to bind the basal and secondary prism planes, whereas it was devoid of the ability to bind the primary prism and some pyramidal planes. Hence, the replacement of specific IBS residues impaired the protein’s binding to different ice planes. These observations suggest that residues T20 and A214 play different roles in basal-plane binding, which might affect the AFP hyperactivity. The T20Y/A214Y double mutant lost the ability to bind the basal, primary prism, and all pyramidal planes, except for the secondary prism planes, as shown in Fig. 2b, panels 5 and 6, similar to the T20Y mutant. Further, the mixture of T20Y and A214Y mutants appeared to cover nearly the entire hemisphere, incompletely recovering the ability to bind to the primary prism plane, as shown in Fig. 2c. This observation, together with the partial recovery of the TH value shown in Fig. 5b, suggests that the two mutants preferably adsorb to discrete ice planes via their IBS, to partially compensate for their defective activity. It has been proposed that in the hyperactive *Col*AFP^20^ and *Tis*AFP8^22^ proteins, the loop region of compound IBS is involved in basal-plane binding. The findings of the current study offer additional experimental evidence in support of this notion. Also, partial recovery of ice-binding ability observed for the mixture of the defective mutants exhibits that both T20 and A214 are required for binding to entire planes of ice crystal while they are proposed to play different roles in basal-plane binding. This view also suggests that the loop and sheet region of compound IBS should adsorb together to the ice plane to exert the original antifreeze activity of *Tis*AFP7. The spacing between the adsorbed AFP molecules on the ice plane was estimated as 70–350 Å for *Tm*AFP-GFP^40^. Here, we assume that *Tis*AFP7 also adsorbs to ice in a similar interval with *Tm*AFP. Also, if we assume that the loop and sheet IBS individually adsorbs to the ice plane in the mixture of the defective mutants, these two IBS might be too distant to cover entire ice planes, which might bring about the partial recovery of ice-binding ability. Therefore, the compound IBS situated in the same molecule should be essential for the full antifreeze activity of *Tis*AFP. From this point of view, we speculate that the loop and sheet region of IBS of *Tis*AFP might simultaneously bind to a target ice plane although the surfaces of the loop and sheet IBS does not make a flat plane and intersect with approximately 150°.

### Network structure of water molecules bound to IBS

In one asymmetric unit, two *Tis*AFP7 molecules were surrounded by 388 and 358 bound water molecules, respectively. The hydrated waters on IBS for each *Tis*AFP7 molecule were organized in a specific manner, and could be superposed, indicating that the structure of the bound water molecules is not affected by crystal packing. Among the approximately 54 waters molecules on IBS (Supplementary Fig. S1), six water molecules were aligned in a small trough formed by the inward-facing hydrophobic residues (Val22, Val213, Val195, Val177, Leu150, and Val126) at the β-sheet region of IBS. The trapped water molecules were situated in a line, at an average interval of 4.6 Å. Typically, the distances between the water molecules 3 and 4, and 5 and 6 were 4.36 Å and 4.99 Å, respectively. The interval between water molecules 3 and 6 was 14.51 Å, which closely matched that between the water molecules in a prism ice plane (4.6 Å and 14.7 Å). The positional similarity with the prism plane may assist *Tis*AFP7 in recognizing the prism plane via the β-sheet region. The trapped water molecules aligning at regular intervals in the IBS trough are also observed in *Tis*AFP6 and *Anp*IBP1a, where they have been proposed to facilitate prism-plane binding^41^. That notion was also supported by the reduced binding of the prism plane by the A214Y mutant (Fig. 2b, panels 3 and 4), where the row of water molecules may be disturbed by eliminating water molecules 2 and 3.

**Figure 6:**
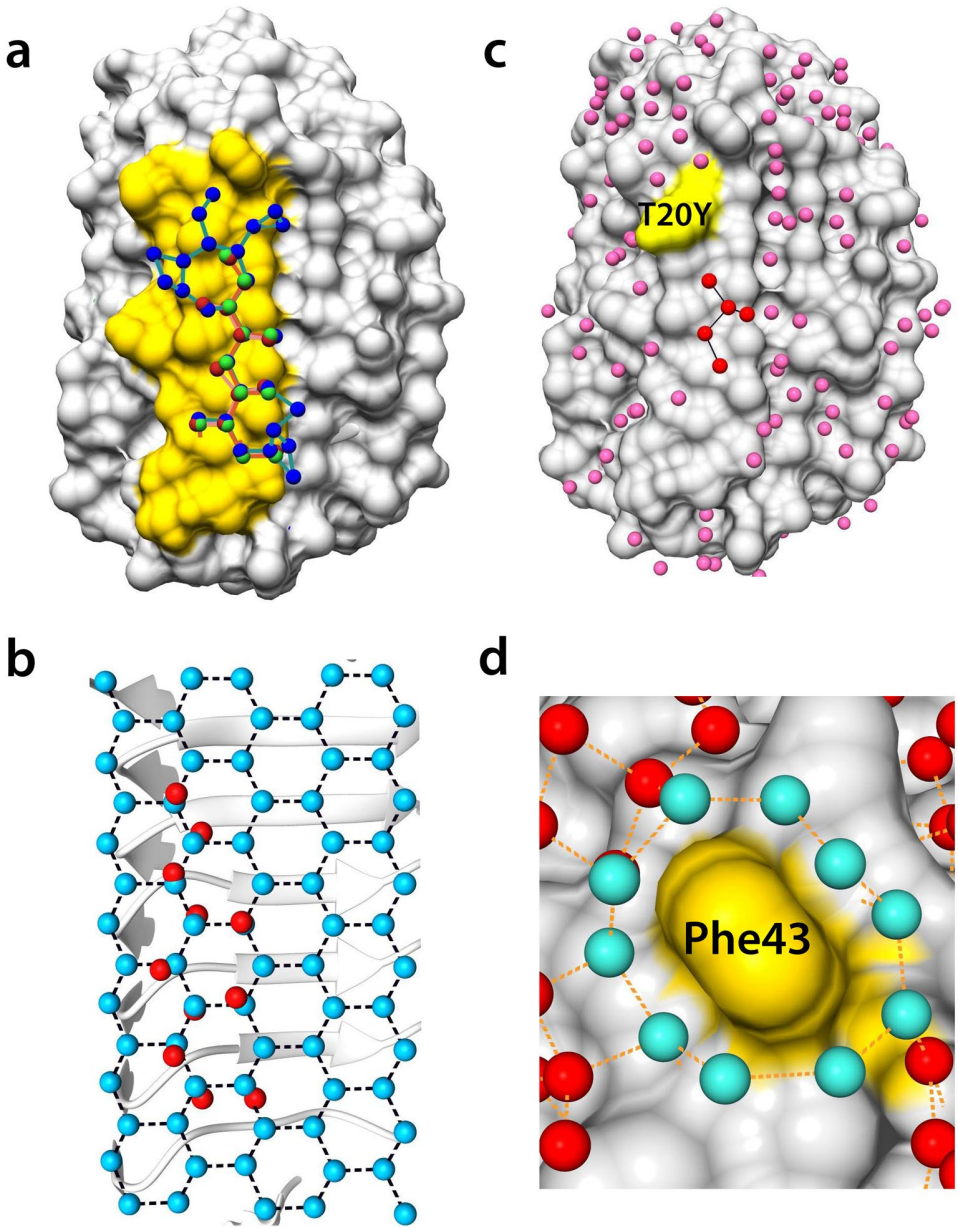
Bound water network and superposition on the basal ice-plane water. (**a**) Surface structure of *Tis*AFP7, with bound water molecules in the loop region of ice-binding site (IBS) colored in yellow. The bound water molecules in *Tis*AFP7, *Tis*AFP8, and *Tis*AFP6 are drawn as spheres in red, blue, and green, respectively. Proximal water molecules, within 3.7 Å, are connected by solid lines. (**b**) Eleven bound water molecules at the IBS loop of *Tis*AFP7 superposed on the basal plane water molecules of an ice crystal with root-mean–squared deviation of 0.77 Å. (**c**) Crystal structure of the T20Y mutant with bound water molecules on its surface. The altered site (Tyr20) is indicated in yellow. IBS water molecules are denoted in red and other bound water molecules in pink. (**d**) Magnified view of Phe43 surrounded by a water ring composed of 10 water molecules drawn in cyan. Each water molecule located within 3.5 Å of each other is connected by a dashed line.

To survey the bound water structure in the IBS loop region in detail, we next compared the structure of *Tis*AFP7 with those of *Tis*AFP6 and *Tis*AFP8. In all these isoforms, 12 water molecules in corresponding positions formed a zigzag-pattern network with branches, shown by red spheres in Fig. 6a. The distance from each water molecule to the proximal water molecule ranged from 2.58 to 3.67 Å, with an average of 2.86 Å (Supplementary Fig. S1c). The angles between three adjacent water molecules were 105–126°, with an average of 116° (Supplementary Fig. S1d). Figure 6b shows the superposition of the water molecule network on a set of ice water molecules in the basal plane (RMSD of 0.77 Å). Close geometrical similarity between the consecutive water molecules on IBS and the ice basal plane appeared to be essential for the ability of *Tis*AFP isoforms to bind the basal plane. However, only *Tis*AFP8 retained a water molecule network extending toward the outer regions of IBS and involving additional water molecules, shown by blue spheres in Fig. 6a. The additional portion of the network was less similar to the ice basal plane than the zigzag waters, but it covered a wider area of IBS than *Tis*AFP7. The comparison of the water network was also illustrated in Supplementary Fig. S4. Water networks have been identified on various AFP structures, including those of the hyperactive *Mp*AFP_RIV^31^ and Maxi^42^ proteins, and that of a moderately active NfeAFP^43^ protein. They form an ice-like structure in the vicinity of IBS, that could merge with the quasi-liquid water layer near the ice surface, as previously proposed^8,44^, thus inducing ice-binding by the proteins.

The water network of *Tis*AFP7 was less extensive than that of *Tis*AFP8. This prompted us to propose that the antifreeze activity of *Tis*AFPs, typically reflecting the affinity toward the ice basal plane, is correlated with the water coverage on IBS via the extended network of water molecules, as observed in the current study. This notion was supported by the truncated water network in the T20Y mutant (Fig. 6d). In the mutant, the bound water molecules near residue 20 were removed by introducing the side chain of Tyr, thus truncating the zigzag pattern.

Further, on the top edge of the IBS loop of *Tis*AFP7, the hydrophobic residue Phe43 was surrounded by 10 water molecules connected in a ring-like manner, as shown in Fig. 6d. The distance between the adjacent water molecules ranged from 2.5 to 3.02 Å, with an average of 2.74 Å. The average distance between the comprising water molecules and the nearest carbon atom of the interior aromatic side chain was 3.4 Å, which implies that these water molecules were constrained to engage in hydrophobic interactions with the Phe43 side chain. In the crystal structure of the hyperactive *Tis*AFP8 protein, no water ring was apparent because of the molecular interactions at Phe43 in the crystal. Nevertheless, a high sequence identity of the IBS residues in the vicinity of residue 43 in *Tis*AFP8 and *Tis*AFP7 might suggest that *Tis*AFP8 coordinates ring water molecules similar to *Tis*AFP7. In addition, in the hyperactive *Col*AFP protein, the corresponding Phe residue is situated in the same position and conformation as that in *Tis*AFP7 and *Tis*AFP8. On the other hand, in the moderately active *Tis*AFP6, Phe is replaced with Ser in this position, and no water ring is observed in the crystal structure, even though IBS is exposed to the solvent area in the *Tis*AFP6 crystal. Based on these observations, we suggest that the hydrophobic hydration on IBS enhances the affinity of *Tis*AFP7 and *Tis*AFP8 for ice, as proposed earlier^45^. The water coverage on IBS for *Tis*AFP isoforms was also assessed as water residence time on the protein surface by using the molecular dynamics (MD) simulation. Table 2 shows water residence time on IBS residues, non-IBS residues, and all residues for *Tis*AFP isoforms. For *Tis*AFP7 and *Tis*AFP8, water residence time on IBS was longer than that on other areas, suggesting that IBS of *Tis*AFP7 and *Tis*AFP8 retain more water molecules. On the other hand, for *Tis*AFP6, non-IBS residues exhibited longer residence time than IBS, which might be attributable to the substituted residues located in the capping head regions, similarly to higher thermal stability estimated by CD measurement. For the bound water molecules on IBS residues, hyperactive *Tis*AFP8 exhibited the longest residence time and *Tis*AFP6 exhibited the shortest, which is the same order for their antifreeze activities. Our current MD calculation offers a simple estimation of the relationship between the water coverage on IBS and its ice plane affinity. Therefore, discussion with the careful calculation should be necessary in the separated report.

**Table 2:** Water residence time for *Tis*AFP isoforms on the molecular surface.

	IBS	Non-IBS	Whole surface
*Tis*AFP7	79.43 ps	68.37 ps	69.90 ps
*Tis*AFP8	85.98 ps	66.93 ps	70.13 ps
*Tis*AFP6	73.16 ps	79.41 ps	78.20 ps

The values were estimated for water molecules making hydrogen bonds with IBS residues (positions 19–25, 38–45, 123–129, 147–153, 174–180, 192–198, and 210–216), and non-IBS residues, and all residues.

Detailed analysis of water-molecule dynamics might be required for further elucidation of the relationship between the bound-water structure and ice-binding property of AFP. The unique hydration structure of surface water of AFP has been reported by utilizing molecular dynamics simulation^30^, terahertz spectroscopy^46^, and sum frequency generation spectroscopy^47^. The current study provides basic insights that can be utilized in these experimental approaches.

To conclude, we here showed that the different activities of *Tis*AFP isoforms are associated with the bound water structure around IBS. The IBS residues act like a platform on a β-helical scaffold, organizing the hydration waters. This is an astounding example of evolution of the antifreeze properties of microbial AFPs, namely, tuning the hydration structure, as a means to adapt to the microbial habitat.

## Materials and methods

### Preparation of *Tis*AFP7 and its mutants

The *Tis*AFP7 (Genbank accession number BAD02892) gene was codon-optimized for *Escherichia coli* and synthesized by GENEWIZ (South Plainfield, NJ, USA). It was then inserted into the expression vector pET38b (Novagen, Madison, WI, USA). The vector was used to transform *E. coli* BL21 (DE3) (Novagen), and the transformants were plated onto Luria–Bertani (LB) agar containing 30.0 μg/ml kanamycin, and incubated at 37 °C overnight. Site-directed mutagenesis of the *Tis*AFP7 gene was performed using the KOD plus mutagenesis kit (Toyobo, Osaka, Japan) with primers listed in Supplementary Table S1, and the mutations were confirmed by sequencing. Protein samples of wild-type *Tis*AFP7 and its mutants were prepared as described by Cheng et al*.*^22^, with a slight modification. Specifically, a bacterial colony was inoculated into 50 ml of LB medium containing 30.0 μg/ml kanamycin, and pre-cultured overnight at 37 °C, with shaking at 130–140 rpm. The culture was then transferred into 1.0 L of fresh LB medium supplemented with 30.0 μg/ml kanamycin, and cultured at 30 °C until the culture OD_600_ reached 0.4–0.8 units. Expression of *Tis*AFP7 was induced by the addition of 0.5 mM isopropyl-β-d-thiogalactopyranoside. The cells were further cultured at 15 °C for 24 h, harvested by centrifuging, and disrupted using high-pressure homogenizer EmulsiFlex-C3 (Avestin Europe, Mannheim, Germany). Cell debris was removed by centrifuging, and the supernatant was dialyzed against 10 mM glycine–HCl (pH 3.0). The dialysate was filtered and loaded onto a cation exchange column Macro-Prep High S (Bio-Rad, Hercules, CA, USA) equilibrated with the same buffer. The adsorbed sample was eluted using a linear gradient of 0–300 mM NaCl. *Tis*AFP7-containing fractions were identified by checking antifreeze activity and sodium dodecyl sulfate–polyacrylamide gel electrophoresis (SDS-PAGE), and were then dialyzed against 25 mM ammonium bicarbonate (ABC) buffer (pH 7.9). The sample was loaded onto an anion exchange column Macro-Prep High Q (Bio-Rad) equilibrated with same buffer. *Tis*AFP7 was collected in the flow-through fraction. The purity of the sample was confirmed by SDS-PAGE and analytical size-exclusion chromatography (SEC) using Sephadex G-75 (Cytiva, Marlborough, MA, USA). Purified *Tis*AFP preparation was concentrated by using an ultrafiltration device Amicon Ultra (MilliporeSigma, Burlington, MA, USA). Protein concentration was determined based on the optical absorbance at 280 nm. Wild-type *Tis*AFP7 eluted as a major fraction after cation-exchange chromatography, and migrated as a single band on SDS-PAGE gel and a single peak after SEC (Supplementary Fig. S2). To examine protein folding and thermal stability of the prepared samples, circular dichroism (CD) spectra were measured for *Tis*AFP7, T20Y, A214Y, T20Y/A214Y mutants, *Tis*AFP6 and *Tis*AFP8. CD spectra were measured on a J-725 CD spectrophotometer (JASCO, Tokyo, Japan) equipped with a temperature-controlled sample holder. *Tis*AFP samples were prepared at 0.25 mg/ml in 10 mM sodium phosphate buffer (pH 7.4) and measured at 4 °C in a quartz cuvette of 1 mm optical path length. As shown in Supplementary Fig. S3a, all the samples except for T20Y/A214Y exhibits CD spectra with similar profiles, which are typical for the β-sheet structure. CD spectra of T20Y/A214Y is not shown in Supplementary Fig. S3a due to a technical reason for a storage device of the raw data while it showed a similar profile to other samples. Subsequently, the thermal stability of each *Tis*AFP sample was observed by monitoring the ellipticity (*θ*) at 220 nm with heating the samples at 0.2 °C interval from 20 to 60 °C at a rate of 1 °C/min. During the temperature scanning, CD spectra were recorded at 4, 20, 30, 40, 50, 60 °C to record the spectral change upon the thermal denaturing process. Typical spectral change is shown in Supplementary Fig. S3b for *Tis*AFP7, which was fully denatured at 60 °C. Fraction of unfolded protein was estimated as $\left({\theta }_{T}-{\theta }_{20^\circ{\rm C} }\right)/\left({\theta }_{60^\circ{\rm C} }-{\theta }_{20^\circ{\rm C} }\right)$ and smoothened by averaged over adjacent values within 0.4 °C, then plotted against temperature (*T*) with 1.0 °C intervals in Supplementary Fig. S3c and d. Denaturation temperature (*T*_m_) was estimated as a midpoint temperature at which 50% of the sample is unfolded. For *Tis*AFP6, the heating process was applied up to 70 °C, and ${\theta }_{70^\circ{\rm C} }$ was adopted instead of ${\theta }_{60^\circ{\rm C} }$ as *Tis*AFP6 exhibited slightly higher thermostability than other isoforms. After the temperature scanning, the sample was cooled back to 4 °C. For all *Tis*AFP samples, the denaturated protein was precipitated in the cuvette even after the cooling back process. Also, CD spectrum observed at 4 °C after heating process revealed the most of samples remain denaturated, as shown in Supplementary Fig. S3b. These observations exhibited that the thermal denaturation of *Tis*AFP is the irreversible process whereas the reversible process was reported for fish AFPs^14,48,49^.

### Photomicroscopic determination of antifreeze activity

The antifreeze activity of protein samples was evaluated by observing ice crystal growth in AFP solutions by using Leica DMLB100 photomicroscope (Leica Microsystems, Wetzlar, Germany) equipped with Linkam THMS600 sample temperature controller (Linkam Scientific Instruments Ltd., Tadworth, Surrey, UK). A color video 3CCD camera (Sony, Tokyo, Japan) was used to capture the images of ice crystals and the temperature of the solution displayed on the screen.

TH of protein solutions was determined as described by Takamichi et al*.*^35^. Briefly, approximately 0.75 μl of AFP sample was placed in a center of a glass capillary tube (10–15 mm long), which was then sealed at both ends with mineral oil. The capillary was placed in a copper holder on the cooling stage. To ensure the thermal conductivity, the clearance gap between the capillary and the stage was filled with 20 μl of ethylene glycol. The sample solution was frozen by cooling to approximately –30 °C, subsequently warmed up to obtain a single particle of ice crystal. The melting point was determined as the temperature at which the ice started to melt. The sample was then incubated for 3 min at 0.05 °C below the *T*_m_. Finally, the ice crystal was cooled at a rate of 0.1 °C/min until rapid growth of the ice crystal was observed. The temperature at which the rapid growth started was recorded as the freezing point, *T*_f_, of the solution. TH measurement was repeated at least three times for each sample at the specified protein concentrations.

### FIPA analysis

To determine the *Tis*AFP7-bound ice plane, FIPA analysis was performed, as described by Garnham et al*.*^50^ and Basu et al*.*^37^. Briefly, a single ice crystal, approximately 40 mm in diameter and 30–40-mm high, was prepared in a suitably sized polyvinyl chloride pipe by gradually growing from a single seed ice. The single crystallinity of the ice crystal was checked by using in-house assembled crossed polarizer. The orientation of the crystallographic *a*-axis was confirmed by the direction of a star shape poked on the ice surface after lyophilization for 10 min. The macroscopic ice crystal was then mounted on a brass cold finger cooled at –1 °C, with the basal or primary prism plane perpendicular to the cold finger. The mounted ice crystal was immersed in chilled milli-Q water and trimmed to a hemispherical shape with 10-mm diameter. Wild-type *Tis*AFP7 was labeled with an orange fluorescent dye [5(6)-TAMRA-X, SE] (Thermo Fisher Scientific, Waltham, MA, USA). The three mutants, *Tis*AFP7 T20Y, A214Y and T20Y/A214Y, were labeled with NHS-Fluorescein (Thermo Fisher Scientific). AFP samples were incubated with the fluorescent dyes in 0.1 M NaHCO_3_ at 25 °C for 3 h, and then dialyzed against water to remove free dye. The mounted ice crystal was grown in 35–40 ml of a solution of fluorescently-labeled AFP (0.007 mg/ml), which was slowly incorporated into the specific ice plane in the hemisphere. The AFP solution was stirred every 20 min. The cooling temperature of the cold finger was gradually lowered to between –7 °C and –8 °C over 3–4 h. The ice hemisphere was detached from the cold finger after achieving a 45-mm diameter, and kept refrigerated at –30 °C for 20–30 min prior to observation. The fluorescence of the ice hemisphere was observed under UV light and photographed.

### Crystal structure analysis of *Tis*AFP7 and T20Y mutant

*Tis*AFP7 and T20Y mutant were crystallized by hanging-drop vapor diffusion method^51^. The initial crystallization conditions were determined by using screening kits, Crystal Screen, Crystal Screen II, and Index (Hampton Research, Aliso Viejo, CA, USA), and Wizard (Rigaku Reagents, Bainbridge Island, WA, USA). For the crystallization trial, the protein solution was concentrated to 30.0 mg/ml for wild-type *Tis*AFP7 and 28.0 mg/ml for the T20Y mutant in milli-Q water. Then, 1 μl of the protein solution was mixed with an equal volume of a mother liquor of the crystallization solution and incubated at 20 °C. A thin plate-like crystal was grown under various conditions, as specified in the kits; all reactions contained polyethylene glycol and polyethylene glycol monomethyl ether 5000. The wild-type *Tis*AFP7 crystal used for the data collection was grown in 0.2 M ammonium sulfate, 0.1 M 2-(*N*-morpholino)ethanesulfonic acid monohydrate (pH 6.5), and 30% (w/v) polyethylene glycol monomethyl ether 5000. For *Tis*AFP7 T20Y, the diffraction data were collected using a crystal grown in 0.2 M MgCl_2_, 0.1 M Tris–HCl (pH 8.5), and 30% (w/v) polyethylene glycol 4000. For diffraction data collection, the crystals were picked up and flash-cooled in liquid nitrogen. The diffraction data for both proteins were collected at the beamline BL-1A in Photon Factory, KEK, Tsukuba, Japan^52^, using 1.1000 Å radiation. The diffraction data were processed using XDS^53^ and CCP4^54^ software suites version 7.0.055 (http://ccp4.ac.uk). The crystal structure of *Tis*AFP7 was determined by the molecular replacement method using PHENIX^55^ and the coordinates of *Tis*AFP8 (Protein Data Bank 5B5H) as a search model. The initial model was examined and corrected by using Coot^56^, and further refined by using PHENIX^55^ and REFMAC5^57^. The crystal structure of T20Y mutant was determined by molecular replacement by using the refined structure of *Tis*AFP7 as a search model and refined by the same procedure as that used for wild-type *Tis*AFP7. To survey the bound water molecules of *Tis*AFPs, which were crystallized in different space groups, the water molecules were rearranged in equivalent positions around the IBS by applying crystallographic symmetry operation of each space group by using Coot^56^.

### Docking with ice plane

To assess the structural similarity of IBS waters and ice waters, the water molecules bound at the IBS loop of *Tis*AFP7 and a set of water molecules comprising the basal plane of ice were superposed. Eleven water molecules at the IBS loop were sampled for superposition with the basal plane of ice crystal. The optimum orientation of the ice plane was determined by using program LSQKAB version 7.0.055^60^ in CCP4 suite, which calculates the presumable minimum distances between bound water molecules and the corresponding ice-plane water molecules. The same calculation was performed for *Tis*AFP8 and *Tis*AFP6. In these analyses, the same numbers of bound water molecules and in positions equivalent to those on *Tis*AFP7 were sampled.

### Molecular dynamics analysis

To compare the hydration network on the molecular surface of *Tis*AFP7, *Tis*AFP8, and TisAFP6, we calculated the residence time for water molecules by molecular dynamics (MD) simulations using the program GROMACS 2020 version 2020.1-Ubuntu-2020.1–1 (http://gromacs.org) ^61^. The crystal structure of each *Tis*AFP isoform was solvated in a simulation box and neutralized by adding counter ions (Na^+^ or Cl^−^). After energy minimization and short position-restrained MD simulation, production MD simulation in 5 ns was performed. Berendsen temperature and pressure coupling were applied, along with the OPLS all-atom force field^62^ and TIP4P water model^63^. The trajectory of the MD simulation was applied to the hbond tool in GROMACS to calculate the residence time of water molecules that are forming hydrogen bonds with the protein surface. The surface of *Tis*AFP was divided into a whole molecule, IBS, and non-IBS to compare the water residence time of each region. The water residence time was estimated as a “forward lifetime” of hydrogen bonds, based on the model by references^64,65^.

## Supplementary Information

Supplementary Information

## Data Availability

The atomic coordinates have been deposited in the Protein Data Bank under the accession numbers ID 7DC5 and 7DDB for the wild-type *Tis*AFP7 and *Tis*AFP7 T20Y, respectively.
