# Supplementary material for: Characterization of microbial antifreeze protein with intermediate activity suggests that a bound-water network is essential for hyperactivity
Source: Sci Rep. 2021 Mar 16;11:5971. doi: 10.1038/s41598-021-85559-x (PMC7966756; doi:10.1038/s41598-021-85559-x)
Supplement: Supplementary file 1 — Supplementary Information [file 41598_2021_85559_MOESM1_ESM.pdf]

Supplementary Information for

Characterization of microbial antifreeze protein with intermediate activity suggests that a bound-water network is essential for hyperactivity

N.M.-Mofiz Uddin Khan[1,2], Tatsuya Arai[1], Sakae Tsuda[1,3,4] and Hidemasa Kondo[1,3,*]

[1]Graduate School of Life Science, Hokkaido University, Sapporo, 060-0810, Japan.

[2]Department of Chemistry, Dhaka University of Engineering and Technology, Gazipur Gazipur, 1700, Bangladesh.

[3]Bioproduction Research Institute, National Institute of Advanced Industrial Science and Technology (AIST), Sapporo, 062-8517, Japan.

[4]OPERANDO Open Innovation Laboratory, National Institute of Advanced Industrial Science and Technology (AIST), Tsukuba, 305-8563, Japan.

[*]Correspondence and requests for materials should be addressed to H.K. (email: h.kondo@aist.go.jp)

2-17-2-1, Tsukisamu-Higashi, Toyohira, Sapporo, Hokkaido 062-8517, Japan.

+81-11-857-8996

+81-11-857-8983 (FAX)

Supplementary Table S1 Sequence of primers used for the construction of *Tis*AFP7 mutants

Protein	Primer sequence
T20Y	5′–**TAT**GCCGTTAGCACCGTTCCGAGA–3′
	5′–GCTGGCCAGAATGGCATAATTACCTG–3′
A214Y	5′–**TAT**CTGCAGAGCGCCACCGTGGTG–3′
	5′–AACGCTGGTCTGGGCCAGAATGCG–3′

The nucleotide positions corresponding to each mutation site are shown in bold.

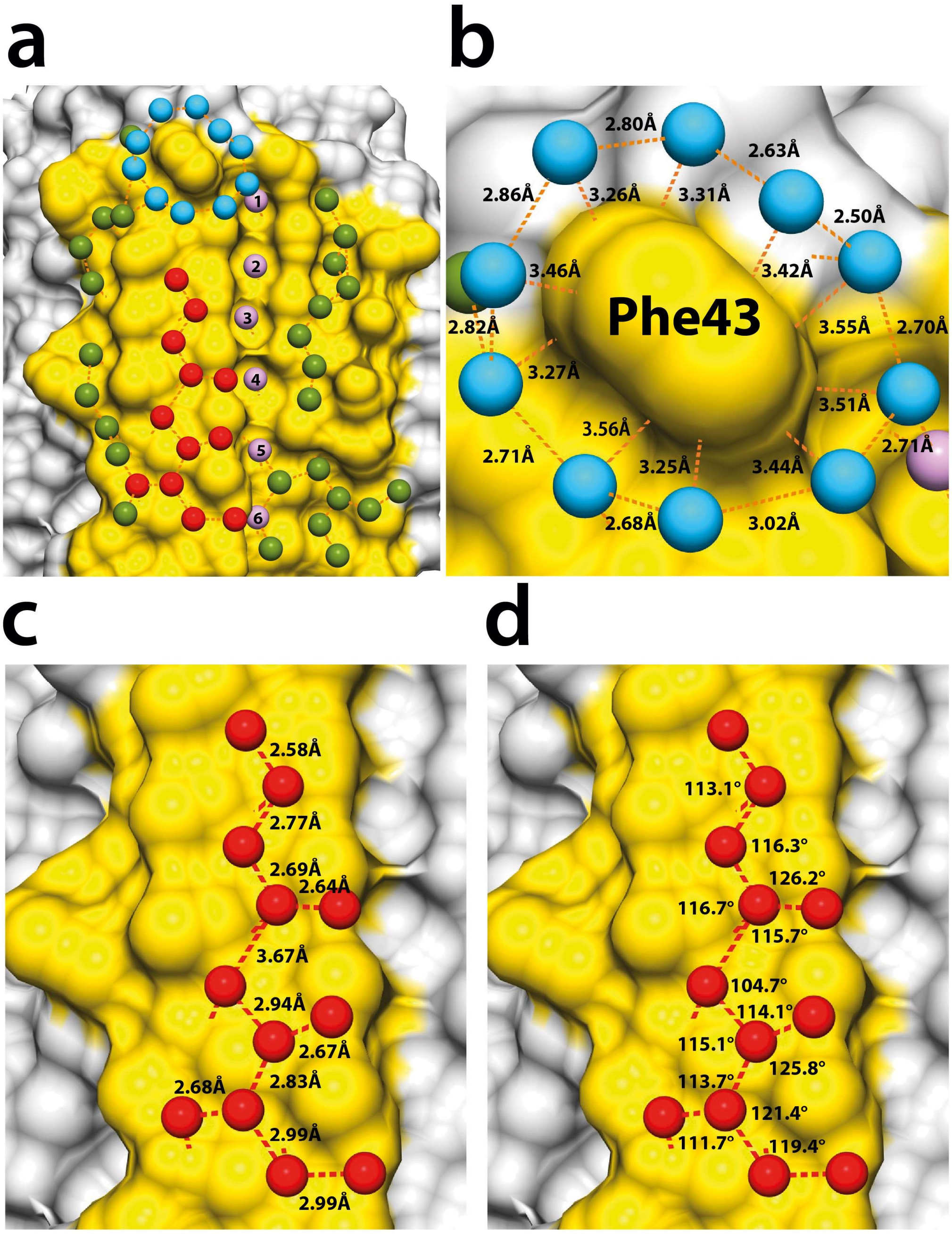

Supplementary Figure S1. Network of bound water molecules on the ice-binding site (IBS) of *Tis*AFP7. (**a**) Overview of the surface area of IBS (denoted in yellow), with the water molecules drawn as spheres. Six water molecules aligned in a trough on IBS are denoted in pink and numbered 1–6. Bound water molecules on IBS loop forming a zigzag pattern are denoted in red. Other water molecules on IBS are denoted in green. (**b**) A water ring surrounding Phe43 side chain, denoted in cyan. Interaction between water molecules and side chain atoms are shown, with the distance between the proximal water molecules and carbon atoms indicated. (**c**) Distances between the zigzag water molecules. (**d**) Angles between three adjacent water molecules.

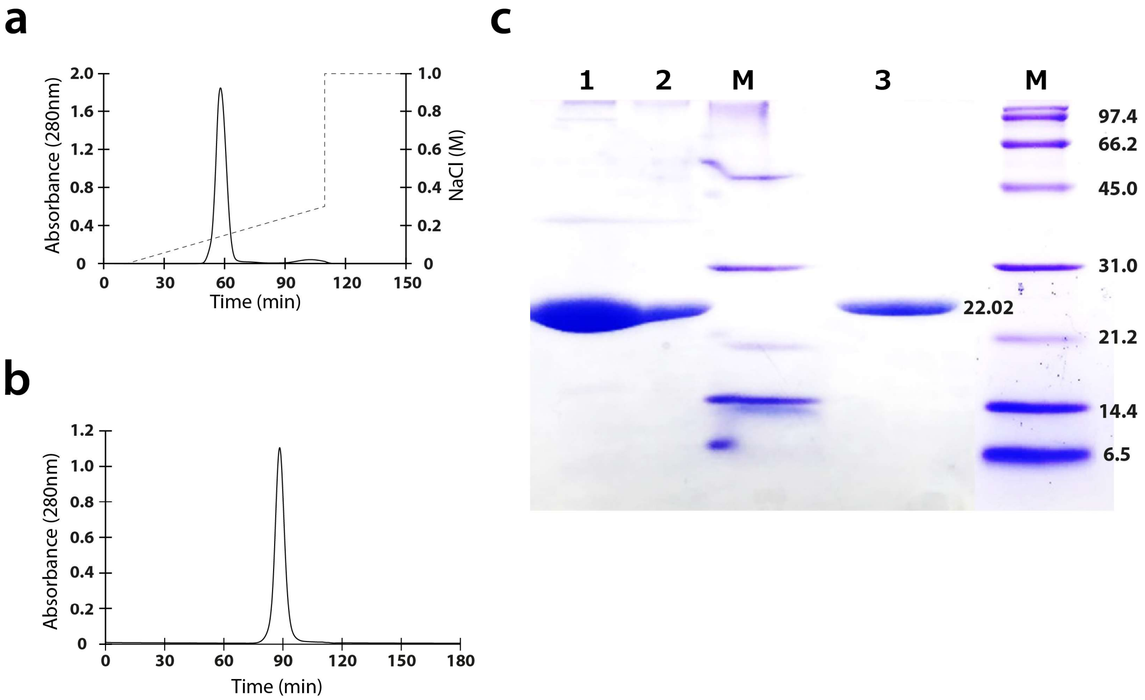

Supplementary Figure S2. Purification of *Tis*AFP7. (**a**) Chromatogram of a cation exchange chromatography. Y-axis on the left side shows the optical absorbance at 280nm. A concentration of NaCl applied to elute the adsorbed sample is indicated in the right y-axis. (**b**) Chromatogram for analytical size-exclusion chromatography for checking the purity of the sample. (**c**) Sodium dodecyl sulfate–polyacrylamide gel electrophoresis of the purified *Tis*AFP7. Lane *1* and *2*, fractions after a cation exchange chromatography. Lane *3*, the flow-through fraction of anion exchange chromatography. *M*, molecular weight standard.

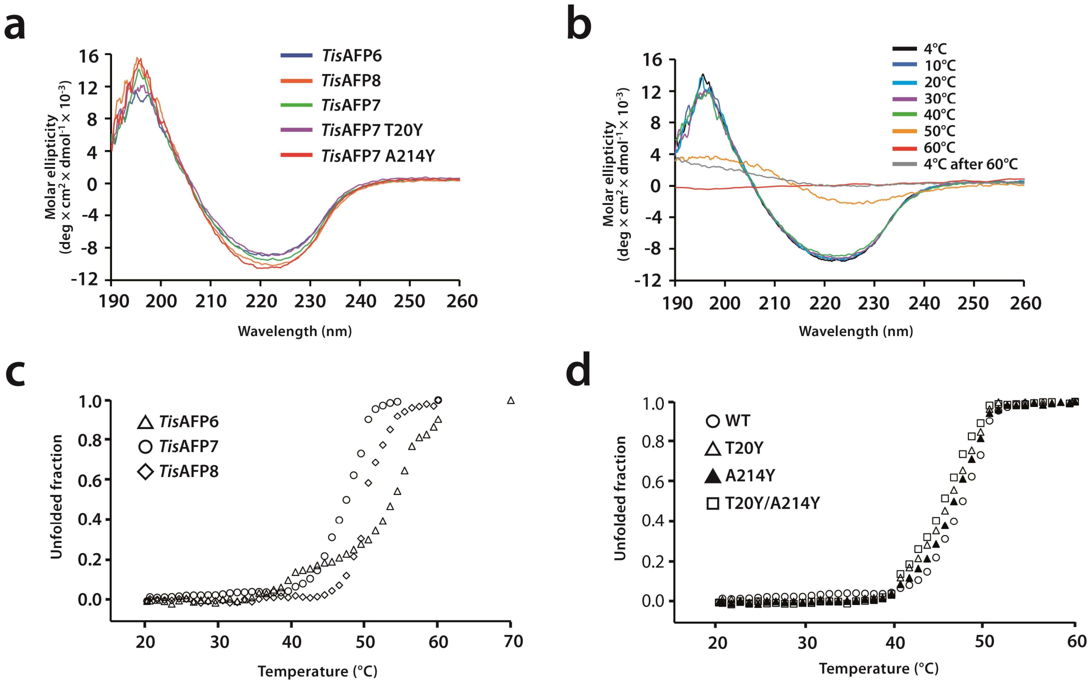

Supplementary Figure S3. Thermal denaturation of *Tis*AFP7, T20Y, A214Y, T20Y/A214Y mutants, *Tis*AFP8 and *Tis*AFP6 estimated by CD spectrometry. (**a**) CD spectra of *Tis*AFP7, its mutants (T20Y, A214Y), *Tis*AFP8, and *Tis*AFP6 observed at 4 °C. (**b**) CD spectra change of TisAFP7 upon heating. Measurement temperatures are indicated in the legend. (**c**) Thermal denaturation profiles of *Tis*AFP7, *Tis*AFP8, and *Tis*AFP6. (**d**) Thermal denaturation profiles of *Tis*AFP7, T20Y, A214Y, and T20Y/A214Y.

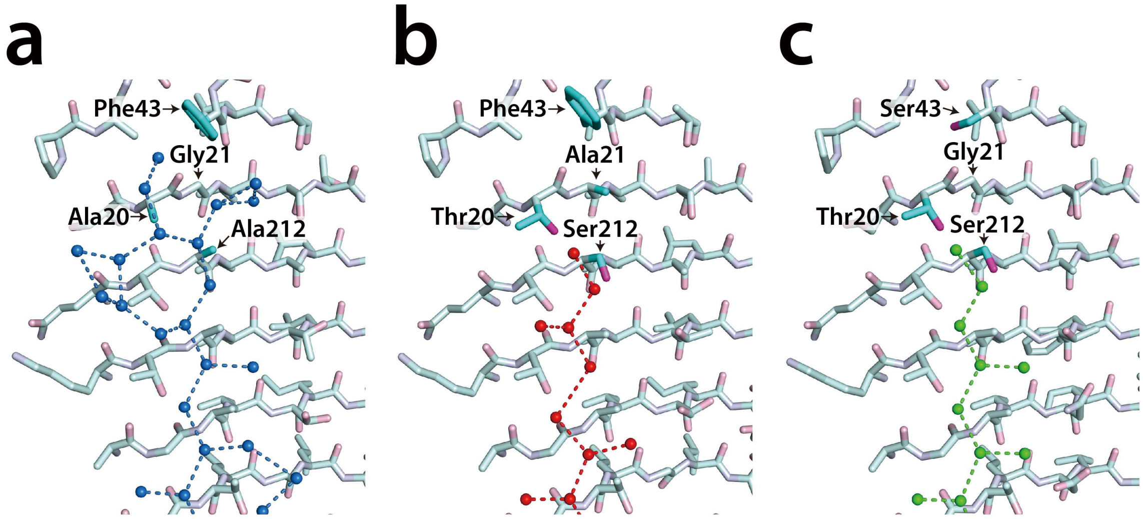

Supplementary Figure S4. The structure of the water network and *Tis*AFP isoforms are aligned for comparison with three isoforms, (**a**) *Tis*AFP8, (**b**) *Tis*AFP7 and (**c**) *Tis*AFP6, viewing from the same orientation. Water molecules that are positioned within 3.7Å from adjacent water are drawn as spheres and connected by the dotted lines. The protein structures are illustrated for the residues located in the ice-binding site as a stick model. The residues that are substituted between the isoforms are indicated by arrows. The figure was prepared using the software open-source PyMOL v. 1.5.0.3 (http://pymol.org)[1].

Reference

1 Schrödinger, LLC The PyMOL Molecular Graphics System, Version 1.5.
